# Adaptive Management: From More Talk to Real Action

**DOI:** 10.1007/s00267-013-0205-7

**Published:** 2013-11-23

**Authors:** Byron K. Williams, Eleanor D. Brown

**Affiliations:** 1 Science and Decisions Center, U.S. Geological Survey, 12201 Sunrise Valley Drive, Reston, VA 20192 USA; 2 Present Address: The Wildlife Society, 5410 Grosvenor Lane, Suite 200, Bethesda, MD 20814 USA

**Keywords:** Adaptive management, Decision analysis, Learning, Uncertainty

## Abstract

The challenges currently facing resource managers are large-scale and complex, and demand new approaches to balance development and conservation goals. One approach that shows considerable promise for addressing these challenges is adaptive management, which by now is broadly seen as a natural, intuitive, and potentially effective way to address decision-making in the face of uncertainties. Yet the concept of adaptive management continues to evolve, and its record of success remains limited. In this article, we present an operational framework for adaptive decision-making, and describe the challenges and opportunities in applying it to real-world problems. We discuss the key elements required for adaptive decision-making, and their integration into an iterative process that highlights and distinguishes technical and social learning. We illustrate the elements and processes of the framework with some successful on-the-ground examples of natural resource management. Finally, we address some of the difficulties in applying learning-based management, and finish with a discussion of future directions and strategic challenges.

## Introduction

The challenges currently facing resource managers are large-scale and complex, and demand new approaches to balance development and conservation needs and goals. There is an increasing urgency to conserve biologic diversity, restore, and rehabilitate damaged ecosystems, adapt to climate change, resolve conflicts in resource allocation, and assess the changing condition of organisms and their habitats. Now more than ever, it is important for resource managers to acquire and use information and knowledge to promote sound management of natural resources.

One approach that shows considerable promise is adaptive management, which by now is broadly seen as a natural, intuitive, and potentially effective way to make decisions in the face of uncertainties. Adaptive decision-making involves the use of management itself to pursue management objectives and simultaneously learn about management consequences.

Though it offers new opportunities to inform decision-making and improve the management of natural resources, the record of success for adaptive management remains limited. More often than not, research and management are treated as separate activities, implemented in the absence of any framework for their integration. Lee’s (1999) observation that adaptive management has been more influential as an idea than a means of gaining insight into managed ecosystems continues to be relevant today; this is evidenced by the fact that even after 40 years of application, there are relatively few success stories (McLain and Lee 1996; Walters 1997; Stankey et al. 2005; Gregory et al. 2006; Williams and Brown 2012).

Our objective in this article is to present a detailed framework for adaptive decision-making, and discuss the challenges and opportunities in its application to real-world problems. We provide a context and definition for adaptive management, recognizing that the concept continues to evolve and will for the foreseeable future. We describe a framework that emphasizes key roles for both technical and social learning, and illustrate the framework with examples that show the successful use of adaptive decision-making for on-the-ground management of renewable natural resources. We address some of the difficulties in the application of learning-based management, and finish with a discussion of future directions and strategic challenges.

## Background and Context

The phrase “adaptive management” first became connected with natural resource management in the late 1970s (Walters and Hilborn 1978; Holling 1978), and since that time the literature on the subject has grown to be truly huge. During the course of that explosive growth, a large number of definitions for adaptive management have been advanced. Published accounts variously emphasize experimentation (Lee 1993), uncertainty (Williams and Johnson 1995), science (Bormann et al. 2007), complexity (Allen and Gould 1986; Ludwig et al. 1993), management adjustments (Lessard 1998; Johnson 1999; Rauscher 1999), monitoring (Allen et al. 2001; Bormann et al. 2007), and stakeholder involvement (Norton 2005). A relatively recent publication by the National Research Council (2004) defines adaptive management as a decision process with “… flexible decision-making that can be adjusted in the face of uncertainties as outcomes from management actions and other events become better understood. Careful monitoring of these outcomes both advances scientific understanding and helps adjust policies or operations as part of an iterative learning process”. In virtually all cases adaptive management is seen as an evolving process that includes learning (the accumulation of understanding over time) and adaptation (the adjustment of management over time). The sequential cycle of learning and adaptation targets better understanding of the resource system, and better management based on that understanding.

Ever since the introduction of adaptive management in natural resources, a critical feature has been the feedback process between learning and decision-making. Thus, learning is seen as contributing to management by helping to inform decision-making, and management contributes to learning by the use of interventions to investigate resources. Management interventions are often described as experimental “treatments” that are implemented according to a management design, but the resulting learning is seen as a means to an end—namely, effective management—and not as an end in itself (Walters 1986). However great the emphasis on learning, the ultimate focus is on management, and learning is valued for its contribution to improve management. Because its focus is on (human) management with its inevitable ecological consequences, adaptive management is almost always framed in terms of linked socio-ecological systems, with the potential for social as well as technical learning.

There is a broad recognition that adaptive management is potentially useful for many (but certainly not all) resource problems (Williams et al. 2009), namely those for which natural resources are responsive to management but uncertainty exists about the impacts of management. Features with which adaptive management typically is associated include the following. (1) The natural resource system being managed is dynamic, with changes over time that occur in response to environmental conditions and management actions, which themselves vary over time. Included here are systems with spatially identified units, such that each unit is subjected to a single intervention implemented at one of a number of different times over the timeframe. Because of the variable timing of the interventions, at any given time learning from the results of earlier interventions can be used to improve management on units treated later. (2) Environmental variation is only partly predictable, and is sometimes unrecognized. Variable environmental conditions induce randomness in biological and ecological processes, which in turn leads to unpredictability in system behaviors. (3) Periodic management interventions influence resource system behaviors either directly or indirectly. Examples include altering system states such as resource size, or influencing ecological processes like mortality and movement, or altering vital rates like reproduction and recruitment rates. (4) Effective management is limited by uncertainty about the nature of resource processes and the influence of management on them. Reducing this uncertainty can lead to improved management, which is the ultimate goal of adaptive management.

## Framework for Adaptive Management

The literature abounds with descriptions of various frameworks for adaptive management (see for example Allen and Gould 1986; Lee 1993; Ludwig et al. 1993; Williams and Johnson 1995; Lessard 1998; Johnson 1999; Rauscher 1999; Allen et al. 2001; Norton 2005; Bormann and Kiester 2004). Some descriptions are fairly simplistic (e.g., monitoring to recognize success, adjustment of management as needed). Sometimes the framework is little more than a call for best management practices, with follow-up tracking to recognize success. Sometimes the focus is on social learning, in which processes of collaborative decision-making are adapted on the basis of results. In some cases social and technical learning are distinguished, and the potential for both is emphasized. A popular framework involves a cyclic process of planning, decision-making, evaluation, and feedback. Different versions may or may not highlight problem formulation, may or may not distinguish between decision-making and implementation, and may or may not emphasize the role of learning. Variation in the description of adaptive management and its implementation can itself be a source of ambiguity and confusion that limits management effectiveness.

**Fig. 1:**
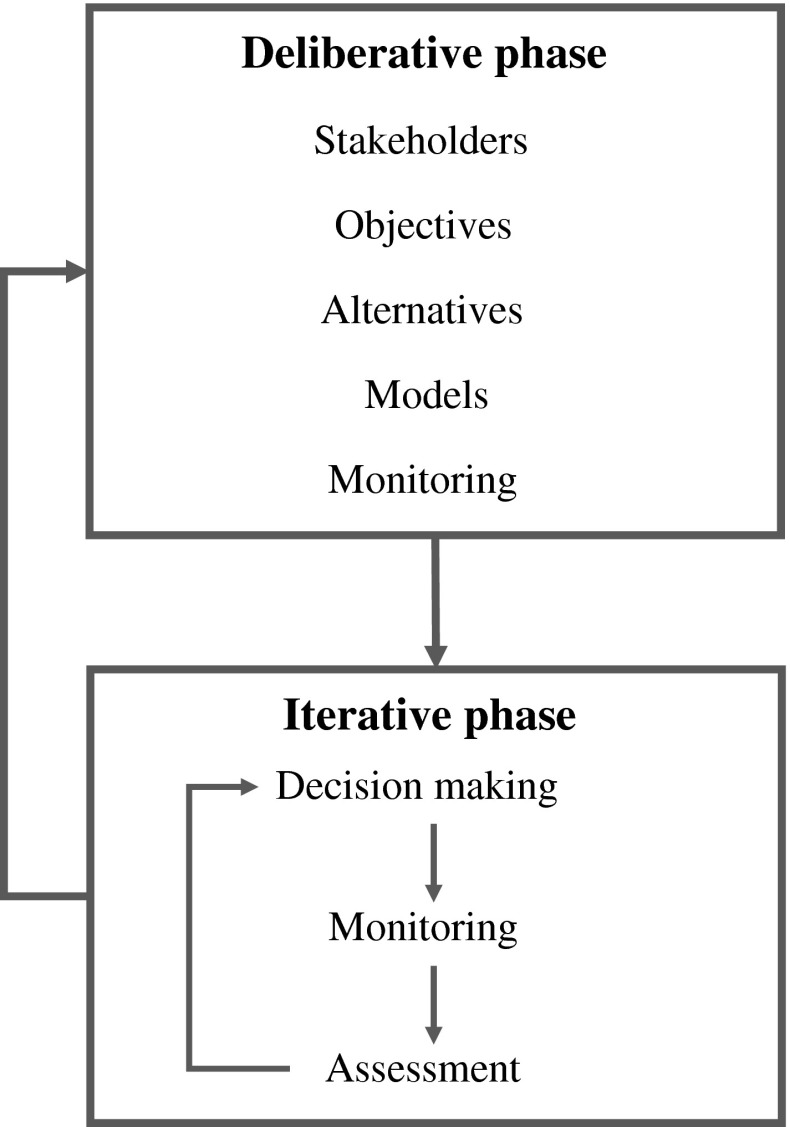
Two-phase learning in adaptive management. Technical learning involves an iterative sequence of decision-making, monitoring, and assessment. Social and institutional learning involves periodic reconsideration of the set-up elements in the deliberative phase

Here we offer a detailed framework for adaptive management that focuses on its essential elements and processes in a two-phase process for both technical and social learning. The framework includes a deliberative or planning phase in which the critical components of adaptive decision-making are formulated, and an iterative decision phase in which the components are linked together in a sequential decision process (Fig. 1). The iterative phase uses the elements of the planning phase in an ongoing cycle of learning about system structure and function, and resource management on the basis of what is learned.

### Deliberative Phase

In the deliberative phase of adaptive management, key elements in the decision-making process are developed and refined, including involvement of stakeholders, determination of objectives, identification of management options, projections of the consequences of management, and design of monitoring protocols. *Stakeholder involvement* is widely recognized as critical in all aspects of adaptive decision-making. Failure to engage important stakeholders, and failure to reach agreement among stakeholders about how to frame a resource problem and identify its objectives and management alternatives, is a common stumbling block that can impede progress and ultimately undermine a project. *Objectives* are essential, as benchmarks against which to compare the potential effects of different management actions and metrics by which to evaluate the effectiveness of management strategies. Feasible and acceptable *management alternatives* are needed for decision-making, learning, and adaptation. The potential consequences of the various different management alternatives are expressed with *predictive models*, which characterize resource changes over time in response to fluctuating environmental conditions and management actions. Models play a critical role as expressions of understanding, as engines of ecological inference, and as indicators of the benefits, costs, and consequences of alternative management strategies. Finally, *monitoring protocols*, including choices about what ecological attributes to monitor and how to monitor them, link closely to the management context and decision-making that motivates the monitoring in the first place. Data collection that is guided by monitoring protocols provides the information needed for both learning and evaluation of management effectiveness. These elements are discussed in greater detail in Williams et al. (2009).

### Iterative Phase

**Fig. 2:**
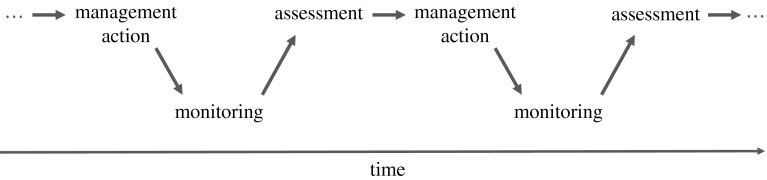
Iterative phase of adaptive management. Management actions are based on objectives, resource status, and understanding. Data from follow-up monitoring are used to assess impacts and update understanding. Results from assessment guide decision-making at the next decision point

In the iterative phase of the framework, the elements in the deliberative phase are folded into a sequential process of decision-making and learning. At any given decision point, *decision-making* identifies actions to be taken based on the current level of understanding and anticipated consequences of management actions on the ground. *Follow*-*up monitoring* provides information to estimate resource status, aid future decision-making, and facilitate evaluation after decisions are made. *Assessment* uses data produced by monitoring to evaluate management effectiveness, identify resource status, and understand resource changes better. *Learning* is promoted by comparing predictions generated by the models and data-based estimates of actual responses, so that understanding gained from monitoring and assessment can provide knowledge for improving future management actions. Figure 2 illustrates an ongoing cycle of decision-making, monitoring, assessment, and feedback that leads gradually to a better understanding of resource dynamics, and a better management strategy based on what is learned.

The actual process of incorporating these elements into adaptive decision-making differs for a sequential approach, in which interventions occur one at a time, and a parallel approach, in which they are implemented simultaneously on different spatial units. Importantly, a sequential approach requires stakeholders to reach agreement about which implementation is to be undertaken at each point in time, whereas a parallel approach allows multiple stakeholder recommendations to be implemented without such an agreement. Of course, in both cases decisions must be made about what interventions to consider, how they are to be implemented, what objectives are to be pursued, and what follow-up monitoring should be undertaken. And in both cases, learning is advanced through the comparison of observed and predicted outcomes, and is folded into future decision-making as it occurs.

### Institutional Learning

A well-designed project provides the opportunity to learn about the decision process as well as the resource system. This learning is obtained by periodically interrupting the cycle of technical learning in the iterative phase to reconsider project objectives, management alternatives, and other elements of the set-up phase (Fig. 1). Reconsideration of these components constitutes an *institutional or social learning* cycle that complements, but differs from, the cycle of technical learning. Learning about institutional arrangements and societal structures and processes requires the development of social capacity and willingness to participate actively in the learning process. A critical consideration is an expanded role for stakeholders, and a more open decision process where learning capacity is valued. In combination, the technical and institutional learning cycles together are referred to as “double-loop” learning (Argyris and Shon 1978).

The need to revisit and adjust the set-up elements of adaptive management often becomes more pressing as management proceeds over time. Stakeholder perspectives and values can shift as management progresses, as previously unanticipated patterns in resource dynamics are exposed and changes in social and cultural values and norms occur. These changes can lead to adjustment of objectives, alternatives, and other set-up elements. In this sense, learning in adaptive management can focus on changes in institutional arrangements and stakeholder values as well as changes in the resource system itself.

**Fig. 3:**
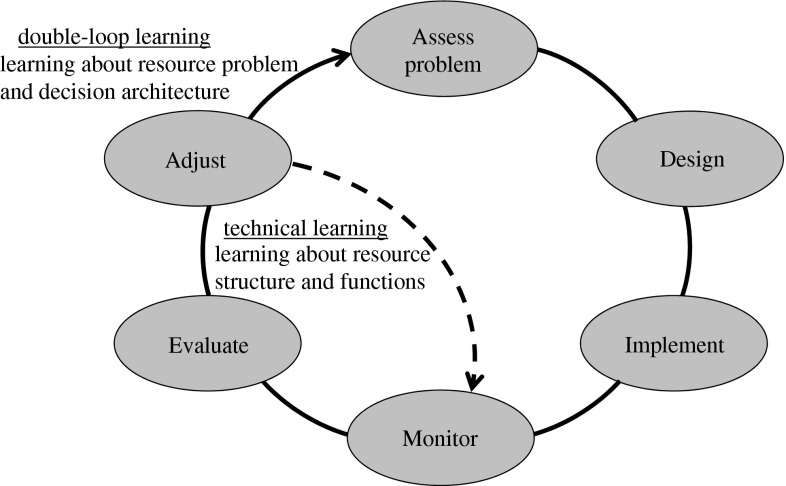
Adaptive management displayed as a cycle, showing technical learning and social/institutional learning. The implementation component refers to implementation of a designed process based on problem assessment, which then is used to initiate technical learning

Adaptive management is often illustrated with a circular diagram (Williams et al. 2009; Gregory et al. 2012) that describes a feedback loop beginning with problem formulation and flowing through decision-making, implementation, evaluation, and feedback into problem formulation (Fig. 3). In the absence of additional structure, such a framework does not distinguish between technical learning and social or institutional learning in a double-loop arrangement. By including an additional feedback loop as in Fig. 3, both kinds of learning can be represented, and the framework can be seen as essentially the same as that presented in Fig. 1.

## Impediments and Alternatives

Adaptive management in the real world of natural resource management continues to evolve, and its value continues to be questioned. If adaptive management makes so much sense in concept, it is reasonable to ask why it has not been implemented more frequently and successfully. The literature on adaptive management points out a number of potential impediments (e.g., McLain and Lee 1996; Walters 1997; Gregory et al. 2006; Williams and Brown 2012). A partial list includes the following.

- A complex decision-making apparatus must be in place or be put in place, and technical expertise and support must be available for people who implement adaptive management. Establishing this type of decision-making framework can involve considerable up-front costs.
- There often is institutional resistance to acknowledging uncertainty. Many managers feel that acknowledging uncertainty is tantamount to an admission that they are not competent.
- Managers often believe that they already know the actions that are needed, and that follow-up monitoring and assessment are unnecessary activities using resources that could be put to better use for conservation on the ground.
- Many people believe that they are already using adaptive management, even when they are not. This occurs most often with projects that involve some ongoing monitoring, in the mistaken belief that monitoring by itself is enough to make a project “adaptive”.
- There is the extreme risk aversion by many managers, which leads to strategies with little or no opportunity for learning.
- Management often is short-sighted, emphasizing near-term gains and losses and devaluing long-term management benefits and costs. If the future is heavily discounted, there is little incentive to use adaptive management to learn how to manage better in the future.
- Stakeholders are not engaged in a meaningful way. Without direct involvement, stakeholders can become disillusioned with management practices, withhold support for a project, or mount legal challenges. Yet many managers are reluctant to include stakeholders meaningfully in decision-making, and thus are prone to insular thinking in the absence of new perspectives and approaches.
- There is a lack of institutional commitment to follow through with the necessary monitoring and assessment after an initial start-up of adaptive decision-making. Monitoring activities include sampling design, data collection and summarization, database management, and data assessment. Many managers are unable or unwilling to continue these activities for extended periods of time.

Given these and other impediments (overlapping jurisdictions; conflicting priorities among scientists, decision makers, and stakeholders), it is not surprising that adaptive management is sometimes viewed with skepticism (McLain and Lee 1996; Walters 1997; Rogers 1998). Several alternative management schemes can be identified (Williams 1997). (1) *Ad hoc management,* which could also be called seat-of-the-pants decision-making, is based on some combination of anecdotal information, the absence of clear management goals, little or no technical foundation for management actions, and inadequate monitoring. This approach can be seen as a primitive variation of trial-and-error management. (2) *Wait*-*and*-*see management*, in which managers refrain from interventions for extended periods of time, is based on the assumption that natural variation will provide enough information to understand the consequences of management. The approach avoids the potential for negative impacts of active management, but does not account for decision-making and the possibility of learning and resource sustainability through management. (3) *Steady*-*state management,* in which managers take their best guess at an optimal resource state, uses management actions in an attempt to eliminate deviations from that state. Above and beyond the obvious problem that there really are no equilibrium conditions in natural resources, steady-state management confounds environmental conditions and management impacts, and thereby limits the opportunity to learn by means of management (see Williams 1997; Gunderson 1999a). This approach also leads to the loss of resilience and an increasing vulnerability to external shocks (Gunderson and Holling 2002). (4) *Conventional state*-*specific management*, which involves the use of explicit objectives and models, is based on an assumption that objectives are appropriate, the resource system is fully observed, and projections of management impacts reflect full understanding. New data are used to track the system’s status, but structural uncertainty and surprise are not represented and accounted for in the assessment of management alternatives. The problem with this approach is that uncertainty is almost always present, though often not explicitly expressed and sometimes not recognized.

Under the right circumstances nonadaptive management is reasonable, for example when there is little uncertainty about what actions to take and what results to expect, or effective monitoring is not possible, or there is no way to feed results of monitoring and assessment back into the management strategy. An adaptive approach can be successful only when the basic requirements for implementation can be met (Williams et al. 2009). When they cannot be met, an alternative approach may be more useful and less costly. But in virtually all cases involving renewable natural resources there is the possibility of unexpected consequences of a management strategy. Even if nonadaptive management is used, it is smart to engage stakeholders actively and maintain enough flexibility in management practice to change the management strategy when the need becomes obvious.

## Examples

Here we describe four applications of adaptive management that exemplify the breadth of applications at different scales and different levels of ecological complexity. We have chosen applications at scales ranging from continental (management of the sport harvest of North American waterfowl [family Anatidae]), to regional (management of the Tallapoosa River in Alabama) to local (visitor management at Denali National Park, commercial take of horseshoe crabs [*Limulus polyphemus*] in Delaware Bay). The management situations vary from a complex aquatic ecosystem involving many stakeholders with contending values and demands (the Tallapoosa), to multiple species and habitats across multiple jurisdictions (the management of North American waterfowl), to the management of one or a few targeted species (golden eagles [*Aquila chrysaetos*] in Denali; red knots [*Calidris canutus rufa*] and horseshoe crabs in Delaware Bay). Management issues include the management of river flows, the protection of migratory birds, the management of visitor disturbance, and the management of commercial exploitation. We note that the nature of the systems represented here requires a sequential approach to adaptive management, in which the system is subjected to a single intervention at each time over the project time frame. This contrasts with situations in which subunits of a system can be treated with different interventions simultaneously.

These applications can be considered as examples of successful adaptive management, in that the full integration of the processes and components of adaptive management produces new knowledge about the respective resource systems, and new knowledge is used to make better management decisions. A commitment to learning-based management, and to the compromise among stakeholders that is necessary, have defused contentiousness and allowed management to move forward in resolving uncertainty and producing positive changes in the resources.

### Tallapoosa River

Extensive hydropower development has altered riverine habitats in the southeastern United States, an important region for freshwater fish and invertebrate diversity. The Tallapoosa River in east central Alabama is a priority area for aquatic conservation, with a native fish assemblage of 57 species, including 5 species endemic to the Tallapoosa River system (Irwin and Freeman 2002). The U.S. Fish and Wildlife Service has been evaluating the relicensing of more than 200 dams in the southeastern US—including the Harris Dam on the Tallapoosa—that are licensed by the Federal Energy Regulatory Commission (FERC). There is a recognized need for new approaches to evaluate dam relicensing, and new strategies to mitigate the impacts of dam operations on aquatic communities. Adaptive management has been used on the Tallapoosa since 2005 to allow for the adjustment of flow management based on what is learned from system responses to water releases. The project is intended to provide a template for incorporating adaptive management and decision support into the broader FERC relicensing process (Irwin and Freeman 2002; Kennedy et al. 2006).

A governance board representing regional and local interests in Harris Dam management has identified objectives and management alternatives for dam and river management, centering on hydropower production, aquatic biodiversity and downstream recreation opportunities (Kennedy et al. 2006; Irwin and Kennedy 2008). Potential conflicts among the objectives identified by stakeholders center on maximizing hydropower versus maximizing aquatic biodiversity and downstream boating opportunities, and tradeoffs among objectives were agreed upon as a starting point for management actions (Irwin and Kennedy 2008). Management alternatives included selections from four alternative daily flow regime options, four alternative “spawning windows” (periods of stable flow), and two boating flow options (Irwin and Kennedy 2008). Modeling of flow regimes and spawning windows was based on different hypotheses about fishes’ dependence on the flow regime and on different hypotheses about recruitment of juvenile fishes during spawning windows in spring and summer (Irwin and Freeman 2002). Monitoring protocols were designed to reduce major uncertainties about the functional relations among flow parameters (e.g., frequency, duration, magnitude, velocity) and fish populations, especially the relationship between periods of stable flow and recruitment of young fishes (Freeman et al. 2001).

The decision-making process was initiated with stakeholders negotiating a starting decision for management actions—an initial flow prescription that consisted of (1) pulsed flows to increase base flow from the dam, thus mimicking natural hydrology in an unregulated reach of the Tallapoosa; (2) periods of stable flow for fish spawning in both spring and summer; and (3) suitable flows for downstream boating in October (Irwin and Kennedy 2008). New information from ongoing monitoring and assessment is used annually to update understanding about fish distributions, hydrologic flows, and recreation capacity. As understanding about the relationships between flow and system responses improves, managers and stakeholders can adjust flow regimes as needed to meet management objectives and ensure conservation of at-risk species. Multiple ecosystem services are considered in the framing of management objectives for the Harris Dam and Tallapoosa River watershed. An adaptive approach offers an opportunity not only to learn about the resource system, but also to learn about the production and valuation of these services as the hydrologic system is managed over time.

### Golden Eagles in Denali

Throughout the Northern Hemisphere, the golden eagle is the pre-eminent diurnal predator of medium-sized birds and mammals in open country. The mountainous regions of Alaska’s Denali National Park support the highest nesting density of golden eagles in North America (Kochert et al. 2002), with undisturbed cliffs for nests that are used over decades or even centuries, and abundant snowshoe hares, ptarmigan, and other prey (McIntyre et al. 2006). Nesting eagles are sensitive to human disturbance, and the National Park Service must limit human presence near nest sites in order to maintain Denali’s eagle population. Eagles may occupy any of nearly 100 potential nesting sites across the northeastern part of the park between March and September during the course of their reproductive cycle of nest repair, egg-laying, and rearing eaglets to independence (McIntyre 2002). This means that a large portion of Denali, a premier national wilderness recreation destination during the summer months, could potentially be off-limits to hiking and other enjoyment of the park. To reconcile the conflicting demands of maximizing recreational access to as much of the park as possible and minimizing disturbance of nesting eagles, the national park uses adaptive management to make annual decisions about whether and how much to limit recreational hiking near nesting areas (Martin et al. 2009, 2011a, b).

Management decision-making focuses on disturbance by hikers, and federal agency managers and scientists worked with the superintendent of Denali National Park to formulate a statement of objectives that includes minimizing the number of sites where hiking is restricted (i.e., no hiking permitted), while maintaining eagle occupancy and reproduction above a specific level (the 20-year average number of territories with successful reproduction) (Martin et al. 2011a, b). Only potential nest sites near the main road through Denali are thought to be exposed to hiker disturbance; therefore, management alternatives involve closure of as many as all of these sites, or as few as none. Three competing models (Martin et al. 2009, 2011a, b) were developed to reflect different hypotheses about the effects of hiking on site occupancy and nest success: (1) no effect, (2) a moderate effect, and (3) a substantial effect. These models are used to generate predictions about future eagle population states as functions of current eagle population state, management actions (restriction of hiking access) and snowshoe hare (prey) abundance. Monitoring protocols require that all potential nest sites are visited each breeding season on multiple occasions until eagles are detected, with a maximum of three visits per site (Martin et al. 2009). Each site at which eagles are detected is visited again in July to assess reproductive success. Data on hare abundance are also collected at each site.

Each year the decisions under consideration are the nest sites at which hiking should be restricted. Objectives, actions, models, and current understanding are used to produce optimal strategies in which the manager incorporates the current condition of the system (eagle occupancy and reproductive success, hare abundance) as evidenced by the most recent monitoring results. An optimal number of sites to be restricted are then identified for each of the possible estimates of eagle and hare “state” (Martin et al. 2009, 2011a, b). Follow-up monitoring involves replicated surveys of all potential nesting sites. Eagle site occupancy and reproductive success are compared each year with the model-based predictions in order to update the credibility weights assigned to three competing models. Each of the three alternative models generates a distinct prediction about the proportion of sites that are expected to be occupied by eagles the next season and the fraction of those at which reproduction is successful (Martin et al. 2011a, b). The changes in credibility measures effectively modify the influence of each model in the decision process, so that models that are better predictors gain more influence. The adaptive management program provides an explicit process for using and monitoring information directly to make management decisions about hiking disturbance. Next steps are consideration of other potential sources of disturbance such as airplane flights for tourists, and future management actions specifying flight paths that limit disturbance.

### Red Knots and Horseshoe Crabs

The sandy beaches of Delaware Bay in Delaware and New Jersey are globally important spawning grounds of Atlantic horseshoe crabs and stopover habitat for long-distance migratory shorebirds such as the red knot, a candidate for listing under the Endangered Species Act (McGowan et al. 2011). The birds stop in Delaware Bay every May to rest and replenish their energy reserves while migrating from wintering grounds in temperate and tropical regions to breeding grounds in the Arctic. They feed on the seasonally superabundant horseshoe crab eggs deposited on the bay’s beaches by millions of crabs that spawn during the lunar tides each spring. Throughout the 1990s a growing and unregulated harvest of horseshoe crabs for use in medical research and as bait in eel and whelk fisheries led to a decline in the numbers of spawning crabs (McGowan et al. 2011). In the late 1990s, monitoring data began to show a major decline in numbers of red knots (McGowan et al. 2011). Shorebird scientists and advocacy groups identified horseshoe crab fishing as the root cause of the red knot decline, while other scientists and horseshoe crab fishermen’s groups argued that red knots are not solely reliant on horseshoe crab eggs for food, and that some other environmental factor must be responsible for their decline. Conservationists wanted a complete cessation of horseshoe crab fishing in the Delaware Bay, while other groups called for more moderate regulations in order to protect the horseshoe crab fishery. Highly variable data, which could be interpreted to support either side in this ongoing argument, resulted in substantial scientific and decision-making uncertainty (McGowan et al. 2011). To facilitate decision-making in this contentious environment, adaptive management was initiated with a goal of identifying a sustainable horseshoe crab harvest strategy that protects red knots and enables learning about how the system functions (McGowan et al. 2009, 2011).

The adaptive management effort has engaged stakeholders in a committee that includes the Atlantic States Marine Fisheries Commission, federal and state fisheries and wildlife agencies, nongovernment organizations, industry and fishermen’s groups, and others. The qualitative statement of objectives expresses the competing resource uses: “Manage harvest of horseshoe crabs in the Delaware Bay to maximize harvest but also to maintain ecosystem integrity and provide adequate stopover habitat for migrating shorebirds” (McGowan et al. 2009). Management alternatives focus on crab harvesting. Possible management alternatives range from a full moratorium on harvesting, to the harvest of up to several hundred thousand crabs, with the potential for differential harvest of male and female crabs (McGowan et al. 2009). Models (McGowan et al. 2011) incorporate three hypotheses about ecological interactions: (1) horseshoe crab spawning abundance has dramatic effects on red knot annual survival and reproductive success, because birds that cannot find enough food during stopover have high mortality and those that do manage to survive the rest of migration that year do not breed; (2) horseshoe crab spawning abundance has a small effect on red knot survival and large effect on reproductive success, because birds that do not gain enough weight during stopover survive the rest of the year with no residual effect, but do not attempt to breed; and (3) horseshoe crab populations have no effect on red knot population dynamics, because some other environmental issue caused the decline of red knots, if in fact the decline truly happened (observed declines may simply be a result of changes in habitat use, or alterations of migratory patterns, or systematic changes in detection rate). The models predict different responses by the red knot population to horseshoe crab harvest. Monitoring protocols (McGowan et al. 2009) involve annual surveys of the population of adult horseshoe crabs with a stratified-transect sampling design during the late summer and fall, after the crabs have spawned and returned to deep waters. Offshore trawling is used to dredge up sampled crabs. Red knot abundance is estimated by mark–recapture techniques, which build on and make use of annual monitoring of red knot weight and body condition.

Each year adaptive dynamic programming techniques provide decision makers with a strategy of optimal harvest actions that are based on the potential abundance of both horseshoe crabs and red knots, and the present degree of understanding about the system. The crab harvest takes place in the summer and fall, after red knot spring migration and crab spawning. Following a harvest, managers monitor populations and compare observed red knot abundance to predictions from the three models to determine which model best represents red knot responses to horseshoe crab harvests. Confidence accumulates over time in the model that makes the most accurate predictions about red knot populations, with the updating of model confidence values by means of Bayes’ rule (McGowan et al. 2011). Institutional learning will occur every few years, when stakeholder groups reconvene to re-evaluate objectives and models (and their underlying hypotheses) in accordance with what has been learned in the iterative phase.

### Adaptive Management of Waterfowl Harvests

Adaptive harvest management was developed to deal explicitly with multiple sources of uncertainty in the regulation of sport waterfowl hunting in North America. Each year, a federally mandated Migratory Bird Regulations Committee develops recommendations for regulating the sport hunting of waterfowl in North America. The committee includes representatives of the U.S. Fish and Wildlife Service (FWS) and the waterfowl flyway councils, with input from nongovernmental organizations and the public. The framework used by the committee is built on an adaptive approach to harvest management, pursuant to an objective of maximizing long-term cumulative harvest for mid-continent mallards (*Anas platyrhynchos*), with an implicit goal of population sustainability and adjustments of harvest utility when population size falls below goals set by the North American Waterfowl Management Plan (Johnson 2011).

**Fig. 4:**
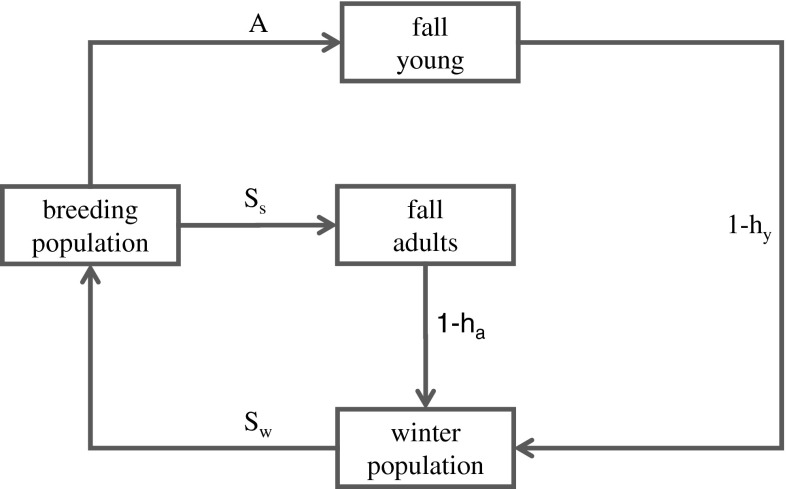
Conceptual model of annual cycle of mallard population dynamics. Model includes survival rates for spring-summer (*S*_s_) and fall-winter (*S*_w_), along with harvest rates for young (*h*_y_) and adults (*h*_a_) and age ratio (*A*) for reproduction/recruitment

Each year the regulatory alternatives under consideration include restrictive, moderate, and liberal strategies, along with a possible closed season. A basic model is used to account for harvest impacts, by representing associations among fall harvest, seasonal survivorship, and spring reproduction (Fig. 4). Different versions of the model incorporate contrasting hypotheses about the impact of harvest on annual survivorship, which describe different functional relations between harvest rates and postharvest survival (Johnson 2011). In addition, contrasting hypotheses about the importance of density dependence in recruitment describe recruitment in terms of spring population size (Cowardin et al. 1985; Greenwood et al. 1995; Johnson 2011). In combination, these hypotheses define four different models, each with its own predictions about harvest impacts and its own measure of confidence that evolves over time. In all four models, reproductive rate is modeled as a function of the number of ponds with water on the Canadian prairie in May, the latter represented as a first-order autoregressive process. Different predictions from each of the four models express uncertainty about population dynamics. Waterfowl monitoring includes surveys (aerial and ground transects) conducted in the principal breeding range of North American ducks twice during the breeding season. Monitoring also includes a large-scale banding program, and surveys of hunters by the FWS to determine hunting activity and the size of the waterfowl harvest (Martin et al. 1979; Smith et al. 1989; Nichols 1991).

The regulatory framework for adaptive harvest management accounts for possible combinations of breeding population size, environmental conditions, and the current level of understanding about population dynamics and responses to harvest. Each year regulations are identified by the Service Regulations Committee, and postdecision monitoring data are used to update biological understanding for the next year. In this way harvest policy changes adaptively over time, as new knowledge is incorporated (Williams 2006).

The objectives, potential harvest strategies, and models projecting the impacts of harvest have changed over time, as new information has been produced and stakeholder perspectives and values have evolved. For example, in recent years, adaptive harvest management has begun to focus on the linkage of harvest regulations and hunter engagement and satisfaction. In fact, waterfowl have become a surrogate for a larger suite of issues—such as habitat conservation and nonconsumptive recreation—that involve a broader array of the ecosystem services that wetlands provide (e.g., flood control, diverse habitats, nutrient recycling). An adaptive approach is being used to address this larger suite of ecosystem services, in efforts to integrate harvest and habitat management more effectively. A big challenge facing harvest management is whether the knowledge and experience gained in its application can be reflected in higher-level structural adjustments when needed. Sorting out these policy and institutional issues will require innovative mechanisms for producing effective dialogue, and new ways of handling disputes within a process that all parties regard as fair.

### Summary

The foregoing examples illustrate that adaptive management can be effective at multiple scales, with various points of ecological focus, different levels of detail, and different kinds of stakeholder engagement. The basic features of an adaptive approach can be seen in all four applications. For example, a key feature is the presence of uncertainty about the resource system and the way it responds to management interventions. Another is the need to engage stakeholders in clarifying the objectives of management, the acceptable management alternatives, and the potential management consequences. All the examples show the components of adaptive management fully integrated in a recurrent cycle of decision-making. Finally, the examples all show the use of management itself to reduce technical and social uncertainty, pursuant to the long-term goal of improved management.

For each of the projects described above, the adaptive management process was designed to allow for a revisitation of the “architecture” of decision-making, including stakeholder involvement, management objectives and alternatives, the models used to project management consequences, and monitoring protocols. For example, the adaptive harvest management project has seen periodic revisions of its objectives, as the values and concerns of stakeholders have evolved. This in turn has led to consideration of new harvest alternatives and changes in the suite of models projecting their consequences. Similarly, the Tallapoosa project was designed from the outset to include a revisitation of its decision-making apparatus through time, with periodic stakeholder reviews of objectives, alternatives, and especially monitoring protocols. The red knot project includes the periodic reconvening of stakeholders every few years, for the express purpose of reconsidering and adjusting both the project objectives and models on the basis of what has been learned.

## Challenges in Adaptive Management

There are a number of large-scale challenges with adaptive management that are tied to changing institutional and environmental conditions. We mention three such challenges here.

### Climate Change

Directional trends in environmental conditions present an important and difficult challenge to management. An obvious example is climate change, as expressed in terms of, e.g., a long-term decrease in average precipitation or an increase in the range of ambient temperatures. Directional change also can be important over shorter periods; many anthropogenic forces exhibit large-scale directional change on shorter time scales than climate change. In either case, the changes have the potential to induce directionality in resource behaviors, i.e., to generate nonstationary resource dynamics (Milly et al. 2008; Nichols et al. 2011).

Nonstationary dynamics are especially challenging for a forward-looking, learning-based approach like adaptive management. Learning about resource processes and the consequences of management on them proceeds through an iterative process of decision-making, follow-up monitoring, and assessment of impacts. The cycle of learning becomes more difficult when the subjects of investigation—the ecological processes that determine resource change—are themselves evolving. One way to address this problem is to track and even model the environmental drivers of change (e.g., Martin et al. 2011a, b), and to use trends in environmental conditions to account for changes in patterns of resource change over time. Another way is to look for limited periods during which resource processes are largely stable, so that learning-based management can be effective. A third approach is to develop environmental scenarios with different patterns of directional change, and design acceptable management strategies that account for uncertainties among the scenarios. Adaptive decision-making then can be used to address uncertainty about which scenario is appropriate, and which strategy therefore should be used (Nichols et al. 2011). Adaptive management can also be used to guide strategies for managing particular adaptations to climate change (e.g., McDonald-Madden et al. 2011).

### Monitoring

The importance of monitoring in adaptive management applications is universally recognized, so much so that some people seem to think that monitoring resource conditions is sufficient in and of itself to make a project “adaptive”. Monitoring certainly plays a critical role by providing the information needed for learning and evaluation of management effectiveness. The value of monitoring in adaptive management springs from its contribution to decision-making, and monitoring protocols should be developed with that in mind.

In fact, monitoring plays multiple roles in adaptive management, by providing information to estimate resource status, underpin decision-making, and facilitate evaluation and learning after decisions are made. It is an ongoing activity, conducted according to the protocols developed in the deliberative or planning phase, and not simply after-the-fact tracking of resource responses in the absence of any capacity to contrast the results against expected responses from different hypotheses. Monitoring can be a highly refined process involving experts and strong controls on field data collection, or it can be a more loosely structured effort perhaps involving a cadre of amateurs who collect the data. In either case, the monitoring program must be carefully designed to ensure a tight connection between management objectives and specific monitoring metrics and protocols, so that the data collected are relevant to assessment, learning, and future decision-making. Attention to the details of who collects data, and how, are critical. Monitoring programs must be designed from the outset with the application of potential results firmly in mind.

Monitoring is often one of the most time-consuming and expensive aspects of adaptive management. During times when budgets are restricted or shrinking, there is always a threat that monitoring will be reduced or eliminated, thereby undercutting the accumulation of knowledge that is needed for evaluation, learning, and decision-making. Because some level of monitoring is almost always required for the smart management of natural resources, it is important to sustain support for tracking and assessment of management consequences. Among other things this means an ongoing stakeholder attention and dedication of resources over the life of a project.

### Organizational Commitment

In spite of frequent assertions that adaptive management is being used, and frequent descriptions of learning as an element of management, there has been only limited progress in promoting a connection between learning and management. Documentation of the institutional structures and processes needed to make an adaptive approach work is also limited (McLain and Lee 1996). For adaptive decision-making, organizations must make a transition from the more traditional “command and control” structure to one that is more inclusive, collaborative, risk tolerant, and flexible (Gunderson 1999b; Stankey et al. 2005). The difficulties of making that transformation, including the sustained commitment of leadership and the staffing of skilled practitioners at the field level, should not be underestimated.

An institution’s recognition of uncertainty as an inherent part of natural resource management is very important. Some hold that adaptive management is not feasible unless the management institutions are willing to embrace uncertainty (Gunderson et al. 1995), which means, among other things, acknowledging different viewpoints and engaging stakeholders with different perspectives in identifying and addressing uncertainties. What is at issue is the structure and context of a learning-oriented organization that can facilitate adaptive decision-making. Attributes of a learning organization include (Senge 1990; Fulmer 2000; Michael 1995): (1) acknowledgment that the world is uncertain; (2) recognition of the importance of training people in the group process skills needed to work effectively in cross-disciplinary teams; (3) positive reinforcement and rewards for experimentation and learning; and (4) recognition that surprises and even crises can be opportunities for learning.

Many observers think that the major challenges in adopting adaptive management are fundamentally institutional (Stankey et al. 2005). Institutions are built on basic premises and long-held beliefs that are deeply embedded in educational systems, laws, policies, and norms of professional behavior (Miller 1999). There is a natural tension between the tendency of large, long-standing organizations to maintain a strong institutional framework for thinking and decision-making, versus adaptive decision-making that relies on collaboration and flexibility, awareness of alternative perspectives, acceptance of uncertainty, and use of participatory decision-making (Gunderson 1999a). Structuring an organization for learning-based management can be hampered by the widespread belief that adaptive management does not constitute a significant departure from past practices, and involves little more than occasionally changing management actions (Stankey and Clark 2006). One consequence is that not enough attention is paid to institutional barriers, and not enough effort is spent on designing organizational structures and processes to accommodate an adaptive style of management. At a minimum, it is necessary to rethink the notions of risk and risk aversion, and establish conditions that encourage and reward learning by individuals.

## Future Directions

Natural resource managers must grapple with critical decisions that bear directly on management of our lands and waters, and our responses to climate change and the continuing alteration of nature by human activities. As we face new opportunities and address new challenges, the principles of adaptive management, including transparency in decision-making and an accounting of both uncertainty and scientific understanding, will be increasingly important. Here we point to some future directions and growth areas for the application of adaptive management.

### Adaptive Management and Planning

**Fig. 5:**
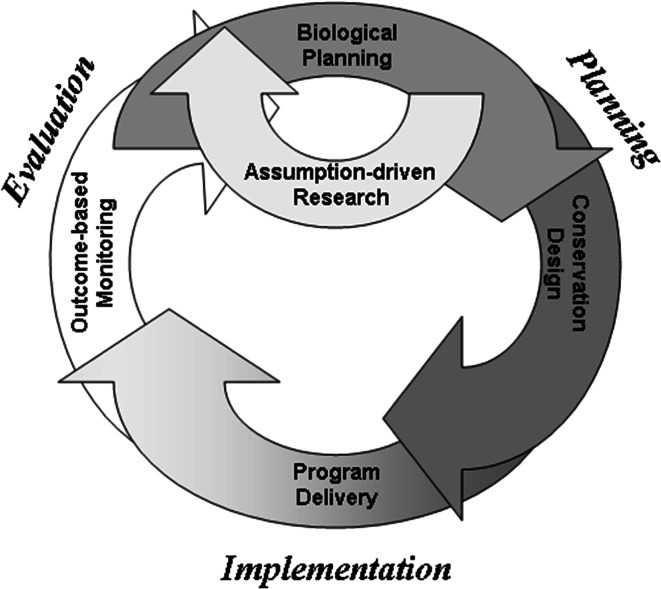
The adaptive cycle in terms of planning, implementation, and evaluation and learning. Planning includes design, assessment and selection of management decisions. Implementation includes management actions on the ground. Evaluation includes social and ecological monitoring as well as analysis and learning

We have characterized adaptive management in this paper in terms of a set-up or deliberative phase in which the elements of adaptive decision-making are developed and refined, and an iterative phase in which those elements are incorporated into a recurrent cycle of decision-making, monitoring, assessment, and learning (Fig. 1). However, adaptive decision-making also can be usefully portrayed as an ongoing process of planning and learning, with the adaptive learning cycle portrayed as a cycle of planning, implementation, tracking, and feedback (e.g., Fig. 5; U.S. Fish and Wildlife Service 2006).

There are natural linkages between these two perspectives. For example, one can recognize the essential elements of strategic planning (the setting of objectives, selection of alternatives, prediction of consequences, tracking of results, etc.) in the deliberative phase in Fig. 1. On the other hand, the elements of strategy implementation such as monitoring, feedback, and adjustment are represented in the iterative phase. Finally, the larger adaptive cycle of institutional learning and adaptation is expressed through double-loop learning. In this sense, adaptive decision-making can be seen as an ongoing cycle of planning, implementation, and learning.

Organizations involved in resource management and conservation engage to varying degrees in both strategic planning and the tracking of results as plans are implemented. Thus, their business practices already involve many of the important elements of adaptive management. A remaining need is to incorporate learning as a fundamental element of strategic planning and implementation, whereby the learning that results from monitoring and assessment is fed back into future planning. By proactively linking plan implementation to plan development through a learning process, the adaptive cycle of learning-based management is completed and becomes standard business practice. A number of important questions need to be addressed in completing the cycle—for example, how to recognize and represent uncertainty, how to track it over time, and how to reduce it efficiently through learning-based management. Nevertheless, the practices currently used for natural resource management have the potential to be incorporated systematically into an adaptive approach.

### New Fields of Application

The practice of adaptive management is not developed evenly in various fields of application. For example, there are many examples in the area of ecology, but few in climate change. In part, this is because the roots of adaptive management are in renewable natural resources, especially biological resources. Applications of adaptive decision-making have been documented for many different biological problems, such as fish and wildlife harvest, insect pest control, endangered species recovery, invasive species control, and wetland management. The examples of adaptive decision-making in biology are extensive and varied, as one might expect of applications developed over the course of more than 40 years.

Conversely, climate change has only recently become a principal focus of conservationists and managers, and is just now maturing as a field of investigation with an agreed-upon conceptual and methodological framework. Under these circumstances it is reasonable to expect fewer examples of adaptive decision-making for climate change mitigation and adaptation. But opportunities for adaptive decision-making are likely to grow rapidly, because systemic environmental change, whether as a manifestation of long-term climate patterns or the result of human-induced landscape alterations, will almost certainly continue well into the future. Environmental change will continue to produce highly uncertain changes in natural resource systems, and resource managers will have to learn about these systems as they are changing. Some initial work has begun on ways to frame this problem in terms of adaptive management (Nichols et al. 2011; Williams and Brown 2012), but much more needs to be done. As the urgency of coping with long-term environmental change increases, there is little doubt that the breadth of adaptive management applications will increase as well.

### Synthesis of Technical and Collaborative Advances

Two broad groups have worked more or less in parallel but independently to develop adaptive management of natural resources. One group focuses on technical issues (models, metrics and propagation of uncertainty, projection of the future consequences of present actions, robust decision-making in the face of uncertainty). The other group focuses on collaboration (institutions, stakeholders, cooperative interactions, elicitation of stakeholder values and perspectives). In this article, we have emphasized the importance of incorporating stakeholder values when identifying objectives, acceptable management alternatives, and models that express stakeholders’ perspectives. On the other hand, it also is important to frame collaboration in terms of science-based decision-making and the technical requirements for the reduction of uncertainty. At present, the collaborative and technical thrusts in adaptive management are being pursued separately, and for the most part researchers, practitioners, and even organizations tend to emphasize either one thrust or the other. The challenge is ultimately to join the two in a more unified vision and process in which each reinforces and strengthens the other.

A number of actions can be taken to facilitate this integration. For example, collaborative and technical organizations can proactively develop bidirectional communications channels. Meetings that now are held separately can be held jointly. Both groups can commit to developing conceptual frameworks that contextualize collaboration in terms of structured decision-making, and structured decision-making in terms of collaboration. Through these and other efforts, the groups can begin to recognize synergies in the partnership for advancing the cause of learning-based resource conservation and management.

### Adaptive Management and Ecosystem Services

Like all strategic approaches to the management of natural resources, adaptive decision-making can have unintended consequences, often for resources that are not the target of the application. The developing field of ecosystem services can contribute to the evaluation of management impacts on the quantity and value of services provided by ecosystems. A potential role for ecosystem services in adaptive management can be seen most clearly in the valuation of ecosystem services, the integration of these values into objectives, and the prediction of changes in ecosystem services and their valuation with models. The connections between adaptive management and ecosystem services need further research, but there are obvious opportunities for collaboration between these important fields of investigation.

### Adaptive Management and Sustainability

Adaptive management emphasizes the importance of accounting for the future consequences of present actions. The idea of change over time is fundamental to adaptive management, whether in terms of changing environmental conditions, repeated adjustment of management strategies, or the use of dynamic models that characterize resource changes. By its very nature, adaptive management requires us to sustain resource structures and functions in order to sustain the ecosystem values that contribute to long-term objectives. In particular, adaptive decision-making has to be flexible enough to respond to the inevitable surprises that arise in resource management, because only then can ecosystems and their values be dependably maintained in the future. Resilience and sustainability have important roles in adaptive decision-making, and their linkages need further examination and development.

## Concluding Remarks

We have described an operational framework for adaptive management, one that accounts for the key components and processes needed for learning-based decision-making. By focusing on uncertainty and using management to reduce it, the application of adaptive management can be expected to improve understanding of the consequences of management, and thereby improve management based on that understanding. We emphasize here the importance of stakeholder involvement in this process, both initially in the design of the management framework and throughout the iterative process. We also emphasize the critical importance of social learning in adaptive management, achieved through periodic revisitation of the “architecture” of decision-making. The tracking and adjustment of evolving stakeholder perspectives, values, and institutional opportunities can be as important as technical learning about the resource system.

We believe that adaptive management holds great promise in expressing and reducing the uncertainties that keep us from managing natural resources effectively. In many cases, the use of management itself in an experimental context may be the only feasible way to gain the understanding needed to improve management. However, the approach does require considerable up-front investment of time and resources to build collaborative networks, to do the hard thinking about system dynamics and management objectives, and to design effective monitoring. As our examples show, the payoff for investing in these activities is improved management over time as understanding increases. Of at least equal importance is the role adaptive management can play in promoting continuing support and engagement of stakeholders, without whose involvement the management of resources can be contentious, litigious, and ineffectual. Better management by means of such a collaborative, objective-driven decision-making process is one important way to promote the conservation of natural resources for future generations.
